# Magnetism of Dendrimer-Coated Gold Nanoparticles: A Size and Functionalization Study

**DOI:** 10.1021/acs.jpcc.1c04213

**Published:** 2021-09-13

**Authors:** José A. Ulloa, Giulia Lorusso, Marco Evangelisti, Agustín Camón, Joaquín Barberá, José L. Serrano

**Affiliations:** † Instituto de Nanociencia y Materiales de Aragón (INMA), CSIC−Universidad de Zaragoza, 50009 Zaragoza, Spain; ‡ Instituto de Nanociencia y Materiales de Aragón (INMA), Departamento de Química Orgánica, Universidad de Zaragoza-CSIC, C/ Pedro Cerbuna 12, 50009 Zaragoza, Spain; § Departamento de Química Orgánica, Facultad de Ciencias Químicas, Universidad de Concepción, 160-C, Calle Edmundo Larenas 129, 4070371 Concepción, Chile; ∥ CNR - Istituto per la Microelettronica e Microsistemi, Unità di Bologna, Via Gobetti 101, 40129 Bologna, Italy

## Abstract

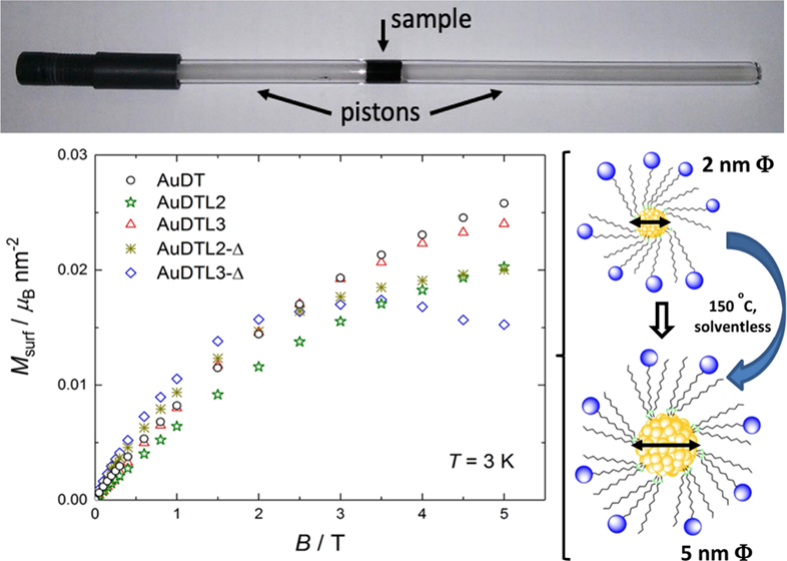

Highly sensitive magnetometry reveals paramagnetism in dendrimer-coated gold nanoparticles. Different types of such nanoparticles, as a result of (i) functionalizing with two distinct Percec-type dendrons, linked to gold via dodecanethiol groups, and (ii) postsynthesis annealing in a solvent-free environment that further promotes their growth have been prepared. Ultimately, for each of the two functionalization configurations, we obtain highly monodisperse and stable nanoparticles of two different sizes, with spherical shape. These characteristics allow singling out the source of the measured paramagnetic signals as exclusively arising from the undercoordinated gold atoms on the surfaces of the nanoparticles. Bulk gold and the functional groups of the ligands contribute only diamagnetically.

## Introduction

It is well known that, for certain metallic materials, size reduction involves variations in the electronic states of the systems and, consequently, in their physical and chemical properties.^1−3^ This fact is particularly remarkable in the electrical, optical, catalytic, and magnetic properties. For example, metals such as Ag, Au, and Cu are diamagnetic in the bulk state, but they show magnetic behavior (ex nihilo magnetism) at the nanoscale (when in the form of nanoparticles or thin films).^4−7^ This phenomenon has been widely studied, mainly in gold nanoparticles, and has opened up a wide range of application possibilities.^8−11^ Logically, considerable effort has been made to explain and interpret the observed properties,^12−15^ many of them related to the chemical coating of the gold nanoparticles.^16−23^

The generation of magnetism in gold at the nanoscopic level, although its origin is not clear,^24^ for some authors is presumably due to the modification of electronic states close to the Fermi level. This is possible through: (i) the hybridization of the Au 5d band with the 3d band of a transition metal,^25^ (ii) size effects such as the increase in the surface/volume ratio,^13,26^ and (iii) the redistribution of charge between the Au 5d orbital and the orbitals of coordinated molecules on the metal surface.^15,21,22^ However, other theories have been published that attempt to explain this phenomenon, such as those based on the generation of orbital currents.^13,27^ The first observations were made by Hori et al.,^26^ who synthesized, by chemical reduction, 2.5 nm **AuDT**-type nanoparticles that present magnetization values of 20 μ_B_. On the other hand, Vager et al.^28^ described the appearance of magnetism when polymeric ligands, based on alanine, were assembled as monolayers in the Au nanoparticles (AuNP’s). The synthesis of 3.5 nm AuNP’s by vapor deposition has also been reported, with magnetization values of 16 μ_B_.^29^ The variables that modify the magnetic behavior and its magnitude in the AuNPs are mainly two, namely, the size dependence, as it is known that a greater surface/volume ratio tends to increase the magnetism,^15,26^ and the functionalization of nanoparticles, which could affect the magnetic character.^21,30^ In the last years, new interesting magnetic properties have been reported as the result of different treatments of gold nanoparticles, for instance, after light irradiation or because of the formation of ensembles.^27,31^

**Figure 1:**
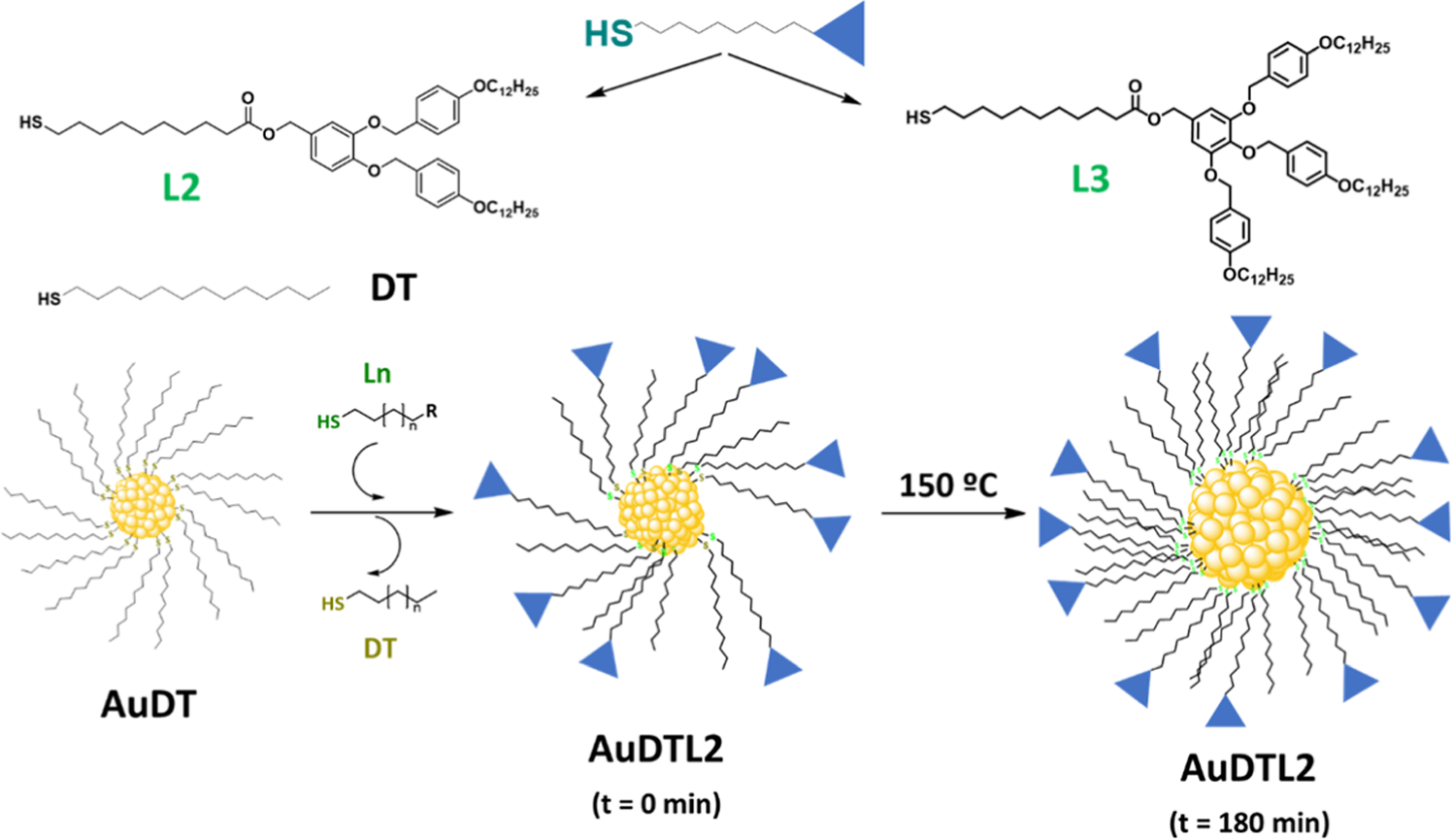
Schematic representation of the ligand exchange reaction (bottom) for dendrimers **L2** and **L3** (top).

We have recently published the formation of dendrimer-coated gold nanoparticles that show low polydispersity. After using a solvent-free process under mild conditions (heating at 150 °C), a controlled growth of these nanoparticles has been produced.^32^ These homogeneous materials allow us to study the influence of both the size and the functionalization of the nanoparticles in the magnetic properties. For this study, we have selected five different types of nanoparticles (see Figure 1): (i) the original gold nanoparticles (**AuDT**) stabilized with dodecanethiol groups, (ii) the nanoparticles (**AuDTL2** and **AuDTL3**) obtained by exchange of ligands of **AuDT** with the **L2** and **L3** Percec-type dendrons,^33^ and (iii) the nanoparticles obtained by thermal treatment of these latter nanoparticles at 150 °C for 180 min (**AuDTL2-Δ** and **AuDTL3-Δ**).

## Materials and Methods

In all cases, these nanoparticles exhibit a spherical structure, as can be proved by scanning electron transmission microscopy (STEM),^32^ and allow their magnetic properties to be parametrized in terms of surface and volume. The masses of the samples employed for the magnetization experiments are 232.3 mg (AuDT), 147.67 mg (AuDTL2), 138.9 mg (AuDTL2-Δ), 44.28 mg (AuDTL3), and 22.55 mg (AuDTL3-Δ).

As can be seen in Table 1, two sizes of nanoparticles have been observed, around 2 nm in diameter for pristine nanoparticles and around 5 nm in diameter for heat-treated nanoparticles. In all cases, the statistical dispersity is around 0.5 nm; these results confirm that the heat treatment simultaneously increases the size of the nanoparticles and their monodispersity. In the SI, the TEM histograms of the **AuDTLn** nanoparticles are represented, both at time zero and after the heat treatment at 180 °C. The TGA studies of the nanoparticles have been carried out in an air atmosphere at a heating rate of 10 °C min^–1^ from room temperature to 800 °C. In all cases, the temperatures at which mass losses occur and total organic mass loss occurs are similar. Representative TGA curves corresponding to Lx nanoparticles are included in the SI section. Besides, using the combination of thermogravimetric measurements and ^1^HNMR data, it is possible to calculate the organic/inorganic content and the proportion of the two organic ligands (dodecanethiol and dendrimers **L2** and **L3**) that bind to the nanoparticle surface (Table 1).

**Table 1:** Size and Composition of the Average Nanoparticles: Mean Diameter (ϕ), Statistical Dispersity (SD), Weight Percentage of the Gold Part (Au% Weight), Percentage of Dodecanethiol Ligand DT (% molar), and Percentage of Dendrimeric Ligands Ln (% molar)

sample	ϕ^a^ (nm)	SD^a^ (nm)	% Au^b^ (% weight)	% DT^c^ (% molar)	% Ln^c^ (% molar)
**AuDT** (*t* = 0)	2.0	0.5	73	100	0
**AuDTL2** (*t* = 0)	2.0	0.5	40	34	66
**AuDTL2-Δ** (*t* = 180)	5.1	0.5	37	33	67
**AuDTL3** (*t* = 0)	2.2	0.5	39	53	47
**AuDTL3-Δ** (*t* = 180)	4.6	0.6	44	53	47

a The measurements corresponding to the mean diameters ϕ (nm) and SD statistical dispersion were obtained by scanning electron transmission microscopy (STEM).

b % weight of the gold part in the hybrid nanoparticle was obtained by thermogravimetric analysis (TGA).

c The proportion of each ligand [% **DT** (dodecanethiol) and % **Ln** (dendronic ligand)] (% molar) was obtained by means of a one-dimensional quantitative experiment of ^1^H NMR.

To rule out the contribution of a possible oxidation state in the gold atoms on the surface of the nanoparticles, XPS studies were carried out. The results indicated that this process does not occur in any case, and no evidence of this gold state was detected in the studied nanoparticles.^32^

**Figure 2:**
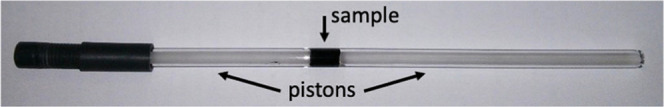
Photograph of the quartz sample holder, with two pistons.

The study of the magnetic behavior of the gold nanoparticles was carried out using a commercial SQUID-based magnetometer, namely, Quantum Design MPMS-XL, equipped with a 5 T magnet. To minimize the background signal and increase the sensitivity of the measurement, a sample holder (SH) designed for this purpose has been used. It consists of a quartz tube with two quartz pistons, one with fixed position and one movable, while the sample is confined between them (see Figure 2). The contribution of the empty sample holder was determined experimentally and was subtracted for each measurement of the nanoparticles. The intrinsic diamagnetism of the sample holder arises from its geometry and is proportional to the volume between the pistons. For each sample studied, the empty sample holder was measured, leaving between the pistons the same space previously occupied by the sample. The size and geometry of the sample holder allowed the use of relatively large sample amounts of ca. 200 mg. Due to the importance of the holder designed and used in these studies, a detailed explanation of its properties as well as its operation is included in Section 1.3 in the SI.

The diamagnetic contributions due to the organic functionalization of the nanoparticles and the gold core were subtracted from each measurement. The estimation of the diamagnetism of the **L2** and **L3** ligands was carried out by direct measurement of the ligands with a magnetometer. For the dodecanethiol ligand (**DT**), this was not possible because the sample is a pasty fluid, and we do not have the necessary accessories to perform these measurements. Therefore, the calculation of its contribution, as well as that of the gold nucleus (naked nanoparticle), was carried out using the data collected in Pascal’s tables.^34^ To subtract the intrinsic diamagnetism of the samples (**AuNP, DT, L2, L3**), the mass percentage values obtained by TGA were considered (see Table 1). Finally, given that the diamagnetic component is temperature-independent, the same contribution is subtracted in each sample at different temperatures.

## Results and Discussion

As we discuss in a previous paper, the data of the TGA and ^1^HNMR measurements gathered in Table 1 show that the number of organic ligands in the pristine **AuDT** nanoparticles is lower than in the cases of **AuDTL2** and **AuDTL3** nanoparticles. Besides, it should be noted that, after increasing the size of the particles by heat treatment, no significant changes were observed in the percentage composition of the ligands. For every sample, larger numbers of ligands than gold atoms were calculated on the basis of a hypothetically spherical shape, hence suggesting full coverage in regard to surface functionalization.^32^ To eliminate as much as possible this excess of ligands, the samples were subjected to a treatment by means of size exclusion chromatography on sephadex as stationary phase.

**Figure 3:**
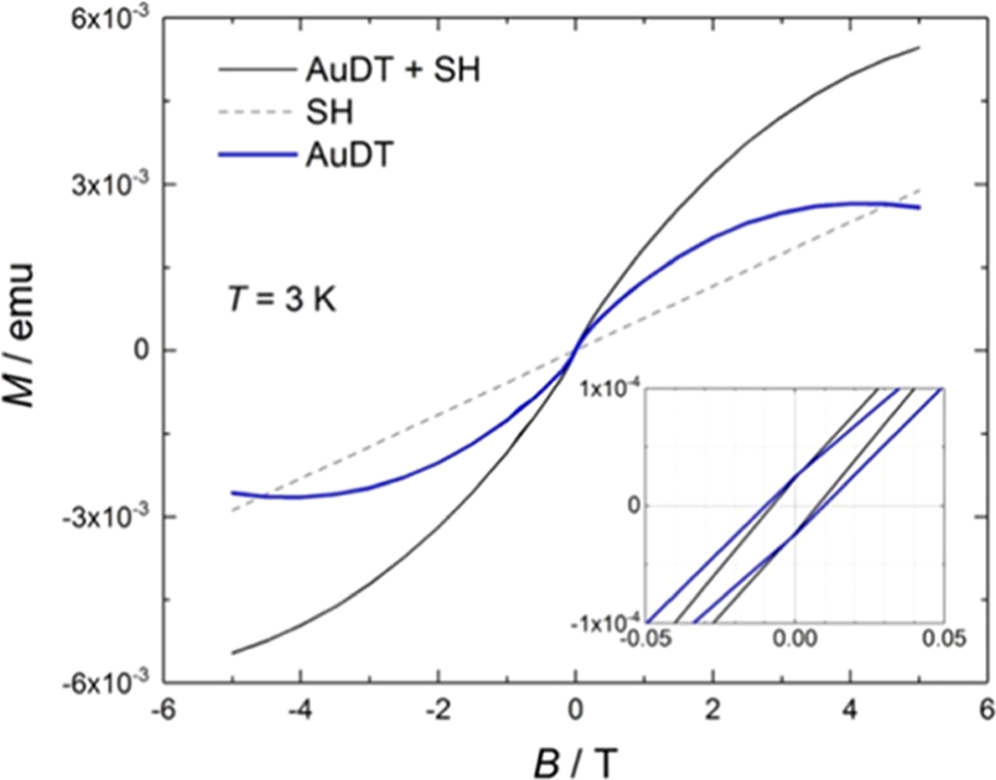
Magnetization versus applied magnetic field for the raw measurements of a 232 mg sample of **AuDT** together with its sample holder (black line) and of the empty sample holder alone (dashed line). The **AuDT** contribution (blue line) is then isolated by mutual subtraction. Inset: Magnification of the hysteresis region.

The hysteresis cycles of magnetization *M* (in emu) versus the applied magnetic field *B* (in T), at a temperature of 3 K, are represented in Figure 3 for: the **AuDT** sample (mass of 232 mg) together with its sample holder (SH) (black line), the empty sample holder (dashed line), and the bare **AuDT** (blue line), as obtained after subtracting the diamagnetic component of **SH**. For **AuDT**, weak ferromagnetism with a coercitive field of 0.01 T is observed (enlargement of Figure 3). We anticipate that this behavior tends to disappear when the organic volume of the samples increases due to the inclusion of the dendritic ligands **L2** and **L3**. It is also important to take into account that the effect of the ligand–nanoparticle interaction is similar in all types of ligands because the interaction is Au–S in all cases. The only difference is the size of the ligand because the L2 and L3 ligands, which have a larger size than the DT ligands, hinder the interaction of the nanoparticles and have a decisive influence on the disappearance of the weak ferromagnetism observed in the AuDT nanoparticles. This result would indicate that the ferromagnetic behavior comes from the interaction between gold nuclei of neighboring nanoparticles.

**Figure 4:**
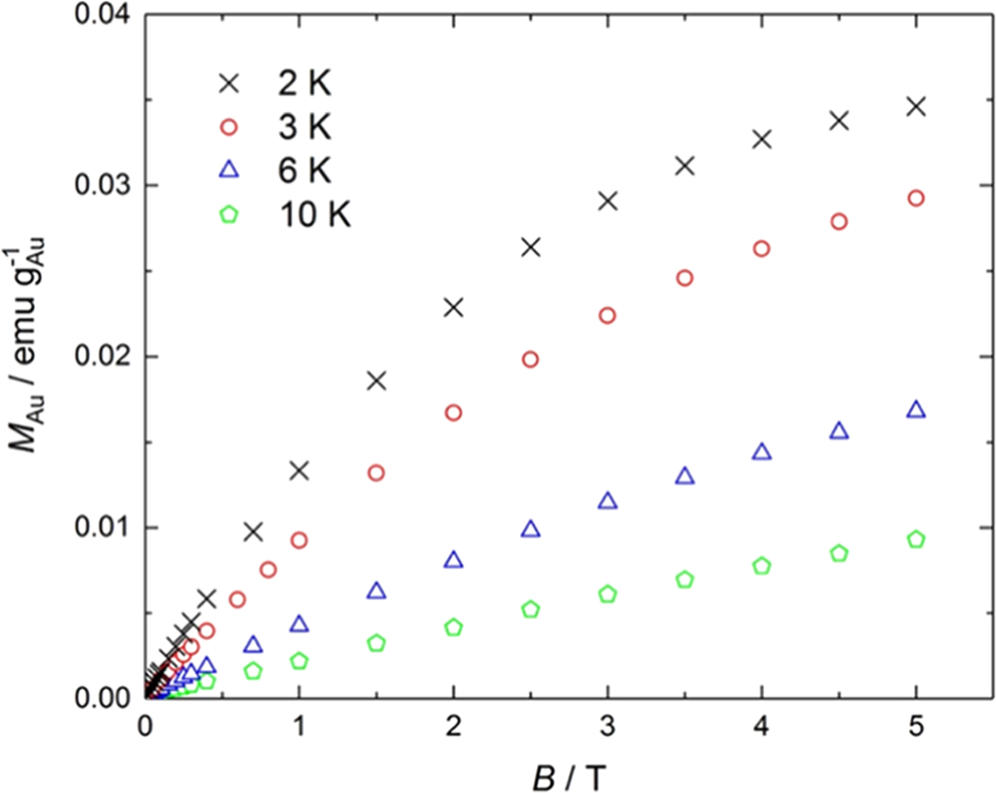
Magnetization per mass of gold, *M*_Au_, versus applied magnetic field, *B*, for the **AuDTL2** nanoparticles (ϕ = 2.0 nm) at temperatures of 2, 3, 6, and 10 K, as indicated.

For all of the samples, measurements of isothermal cycles of magnetization versus applied magnetic field (*B* from −5 to 5 T) were carried out at several temperatures. As a representative example, Figure 4 shows the magnetization curves collected at 2, 3, 6, and 10 K for the **AuDTL2** nanoparticles (ϕ = 2.0 nm), after subtraction of the diamagnetic contributions due to the sample holder, gold core, and organic functionalization. The data are normalized by the mass of gold. For clarity, only results for *B* > 0 are represented. As can be seen, the magnetization increases as the applied field increases and the temperature decreases. Besides, no hysteresis is observed, indicating paramagnetic behavior.

**Figure 5:**
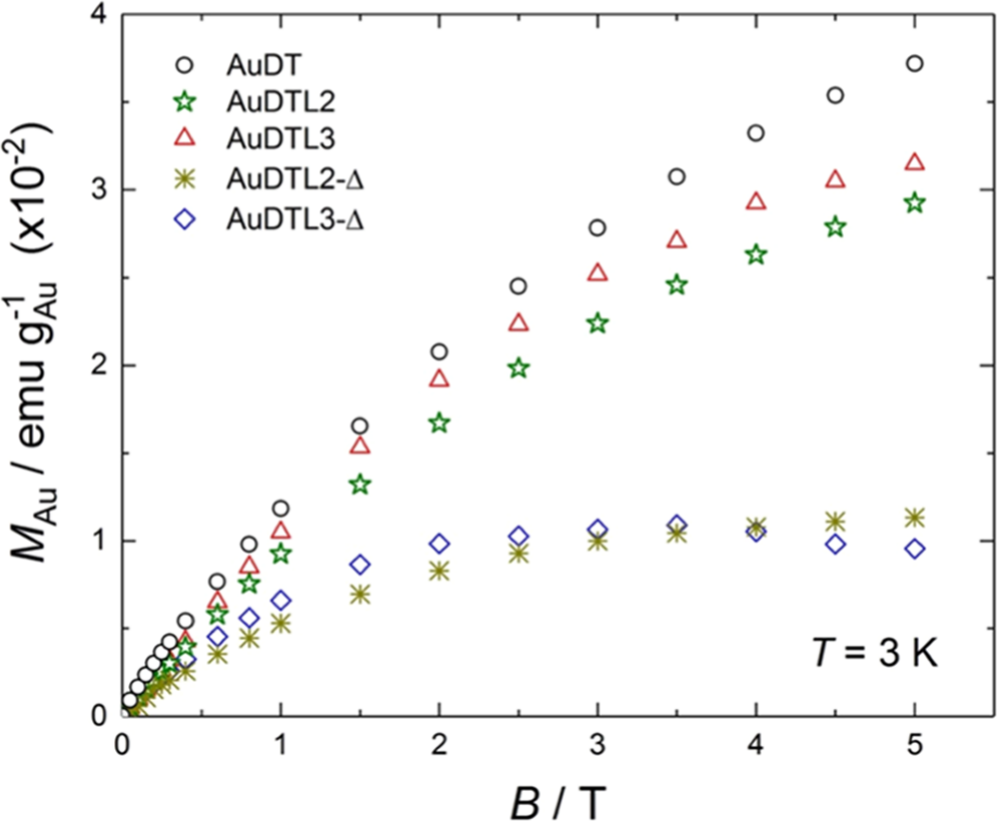
Magnetization per mass of gold *M*_Au_ versus applied magnetic field *B* for **AuDT** (ϕ = 2.0 nm), **AuDTL2** (2.0 nm), **AuDTL3** (2.2 nm), **AuDTL2-Δ** (5.1 nm), and **AuDTL3-Δ** (4.6 nm), as labeled, measured at the temperature of 3 K.

**Figure 6:**
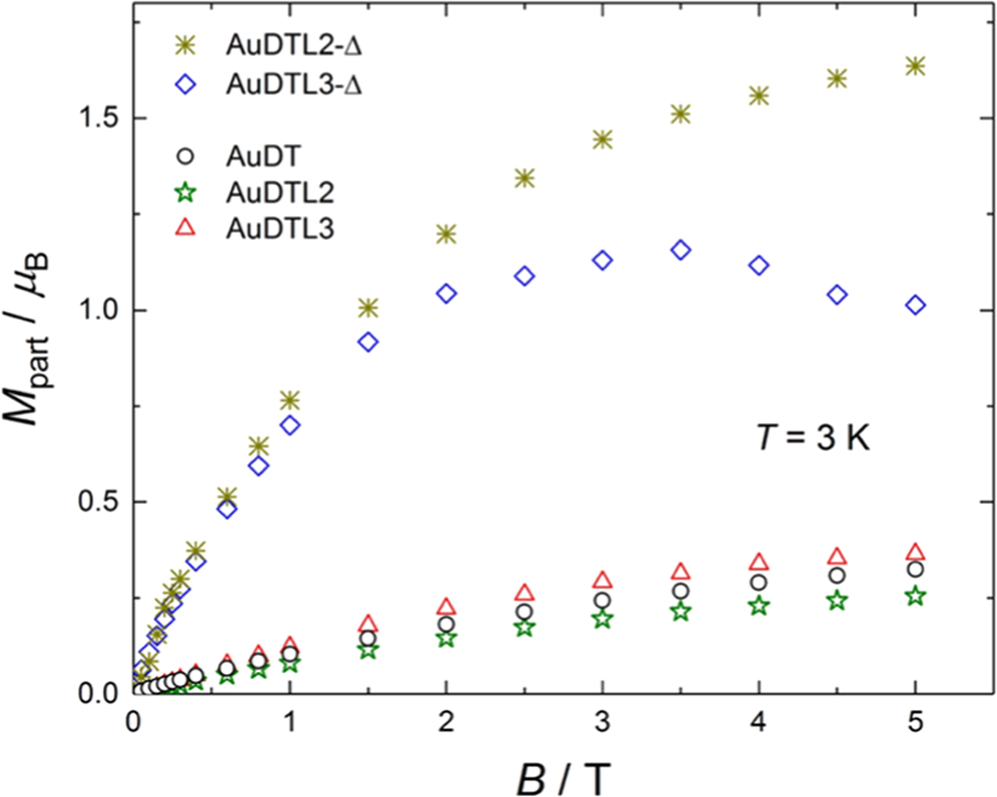
Magnetization per particle *M*_part_ versus applied magnetic field *B* for the particles **AuDT** (ϕ = 2.0 nm), **AuDTL2** (2.0 nm), **AuDTL3** (2.2 nm), **AuDTL2-Δ** (5.1 nm), and **AuDTL3-Δ** (4.6 nm), measured at the temperature of 3 K.

**Figure 7:**
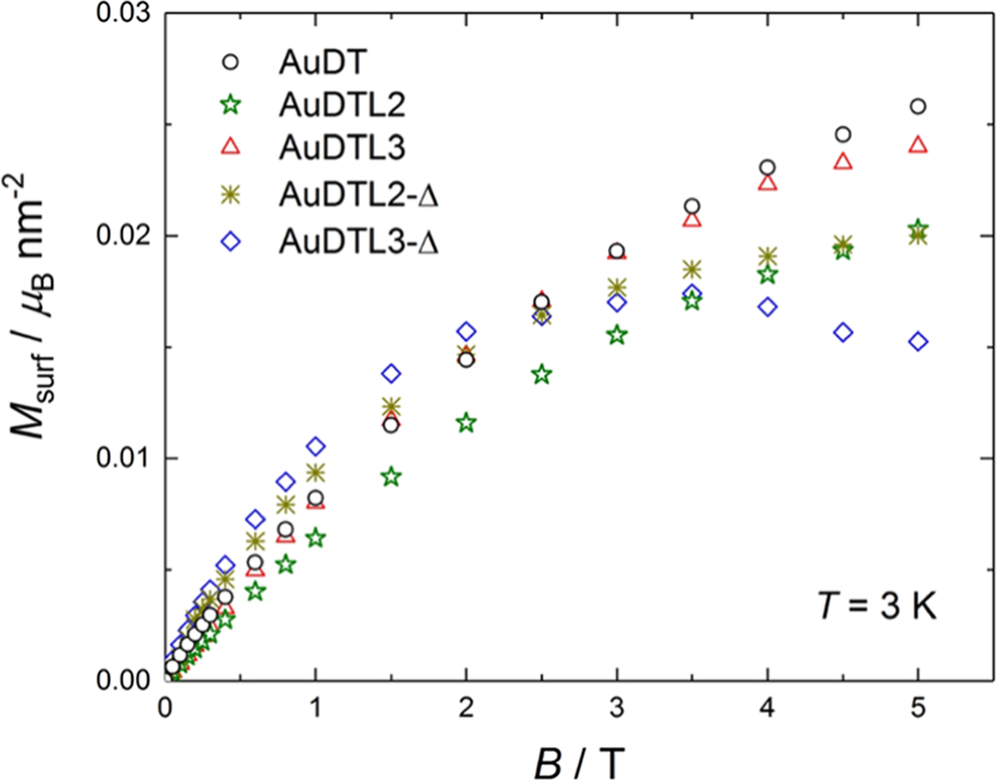
Magnetization per gold surface M_surf_ versus applied magnetic field *B* for **AuDT** (ϕ = 2.0 nm), **AuDTL2** (2.0 nm), **AuDTL3** (2.2 nm), **AuDTL2-Δ** (5.1 nm), and **AuDTL3-Δ** (4.6 nm), measured at the temperature of 3 K.

For direct comparisons, we collect together the data of all of the samples at the representative temperature of 3 K and normalized, respectively, by: mass of gold (Figure 5), particle (Figure 6), and average surface unit of the particles (Figure 7). All diamagnetic contributions were subtracted, as described above.

Figure 5 shows that the magnetization normalized per mass of gold follows two different trends depending on the diameter of the nanoparticles. Specifically, the nanoparticles with ca. 5 nm diameter reach a relatively low magnetization of 1.0(±0.1) × 10^–2^ emu·g_Au_^–1^, while the nanoparticles with ca. 2.0 nm diameter reach a much larger magnetization of 3.3(±0.4) × 10^–2^ emu·g_Au_^–1^, both at *B* = 5 T.

By looking at the magnetization per particle (Figure 6), we obtain a similar, but opposite, behavior: the nanoparticles with ca. 5 nm diameter reach a relatively high magnetization of 1.3(±0.3) μ_B_, while the nanoparticles with ca. 2.0 nm diameter reach a much smaller magnetization of 0.3(±0.1) μ_B_, both at *B* = 5 T. In this figure, due to the increase in the values of the large nanoparticles, a deviation of the values for the **AuDTL3-Δ** nanoparticles is observed at high magnetic fields. This deviation could be explained taking into account the lower weight of the sample as the signal-to-noise ratio increases. However, the trend of the curve is clear and coincides with the results observed in the case of **AuDTL2-Δ**.

These results are consistent with surface atoms being the only source of magnetism for gold nanoparticles.^13,23^ Considering an ideally spherical particle, the volume of the magnetic shell at the surface amounts to πϕ^2^δϕ, where ϕ and δϕ are the particle diameter and magnetic shell thickness, respectively. Assuming δϕ thin and not depending on particle size, by increasing the particle diameter from ca. 2 to 5 nm, the magnetic moment per particle should increase proportionally to the change in volume of the magnetic shell, that is, by a factor of ca. (5/2)^2^ = 6.25, which is consistent with the experimental observation (Figure 6). By these simple arguments, *M*_Au_ should decrease with increasing ϕ, proportionally to the change in πϕ^2^δϕ/(πϕ^3^/6) = 6ϕ – 1δϕ, where πϕ^3^/6 is the whole volume of the Au particle. Therefore, under the same circumstances of increasing the particle diameter from ca. 2 to 5 nm, *M*_Au_ should decrease by a factor of ca. 2/5, in agreement with observation (Figure 5).

To finally verify the influence of the number and type of ligands, and their interaction with the gold atoms, the magnetization data are normalized in Figure 7 by the gold surface *M*_surf_, assuming a spherical shape of the nanoparticles. As can be seen, there is no significant variation in magnetization for all of the cases considered, that is, in these types of nanoparticles, *M*_surf_ does not depend either on the type (**L2** or **L3**) or on the number of ligands (66% **L2** vs 47% **L3**), contrary to that reported in the literature.^17,26^ We must therefore conclude that the ligands, while facilitating the surface gold magnetism through Au–S bonding, contribute only diamagnetically to the overall summation.

The results found in this article coincide with those reported by other authors who attribute the paramagnetism observed in the Au nanoparticles to the effects of the gold atoms on the surface.^4,5^ However, as stated in the two cited reviews,^4,5^ other phenomena could influence these properties. Thus, recently, some authors have reported the influence of the polarity of the ligand bound to the nanoparticle on its magnetic properties.^16^ However, in our case, this effect could not be studied because all of the ligand bonds are Au–S. This union for some authors, as in our case, is fundamental in the appearance of the magnetic properties in the particles.^7,30^

## Conclusions

We have reported highly sensitive magnetization measurements for gold nanoparticles with spherical shape and diameters of ca. 2 and 5 nm. They are stabilized with dodecanethiol groups solely or also with larger dendritic ligands. We have employed a specifically designed quartz tube sample holder in a SQUID-based magnetometer, which has allowed us to discriminate the source of paramagnetism in the dendrimer-coated nanoparticles, as not coming from bulk gold or ligands, but exclusively from the gold atoms at the metal surface.

Weak ferromagnetic behavior was observed for the nanoparticles bearing the dodecanethiol groups solely, but not for the nanoparticles with the larger dendritic ligands. This result suggests that the ferromagnetic behavior comes from the interaction between gold nuclei of neighboring nanoparticles.

## Supporting Information Available

NP’s micrographs and size distribution histograms of the nanoparticles; TGA data analysis proportions of inorganic/metallic core and organic proportion in each sample; holder design, properties and operation (PDF)
