# Supplementary material for: Magnetism of Dendrimer-Coated Gold Nanoparticles: A Size and Functionalization Study
Source: J Phys Chem C Nanomater Interfaces. 2021 Sep 13;125(37):20482–7. doi: 10.1021/acs.jpcc.1c04213 (PMC9236199; doi:10.1021/acs.jpcc.1c04213)
Supplement: Supplementary file 1 — jp1c04213_si_001.pdf [file jp1c04213_si_001.pdf]

Supporting Information

Magnetism of dendrimer-coated gold nanoparticles: A size and functionalization study.

José A. Ulloa[#,γ], Giulia Lorusso[#,ϕ,χ], Marco Evangelisti [ϕ], Agustín Camón[,ϕ], Joaquín Barberá[//] and José L. Serrano*[,//]*

[#] Authors with identical symbol contributed equally.
[ϕ] Instituto de Nanociencia y Materiales de Aragón (INMA), CSIC–Universidad de Zaragoza, 50009 Zaragoza, Spain
[//] Instituto de Nanociencia y Materiales de Aragón (INMA), Departamento de Química Orgánica, Universidad de Zaragoza-CSIC, C/ Pedro Cerbuna 12, 50009 Zaragoza, Spain.
[γ] Departamento de Química Orgánica, Facultad de Ciencias Químicas, Universidad de Concepción, Casilla 160-C Concepción, Chile
[χ] CNR - Instituto per la Microelettronica e Microsistemi, Unità di Bologna, Via Gobetti 101, 40129 Bologna, Italy

CONTENTS

1.1 TEM studies of AuDTLn

1.2 TGA studies of AuDTLn

1.3 Sample holder design and properties

1.1 TEM studies of AuDTLn.

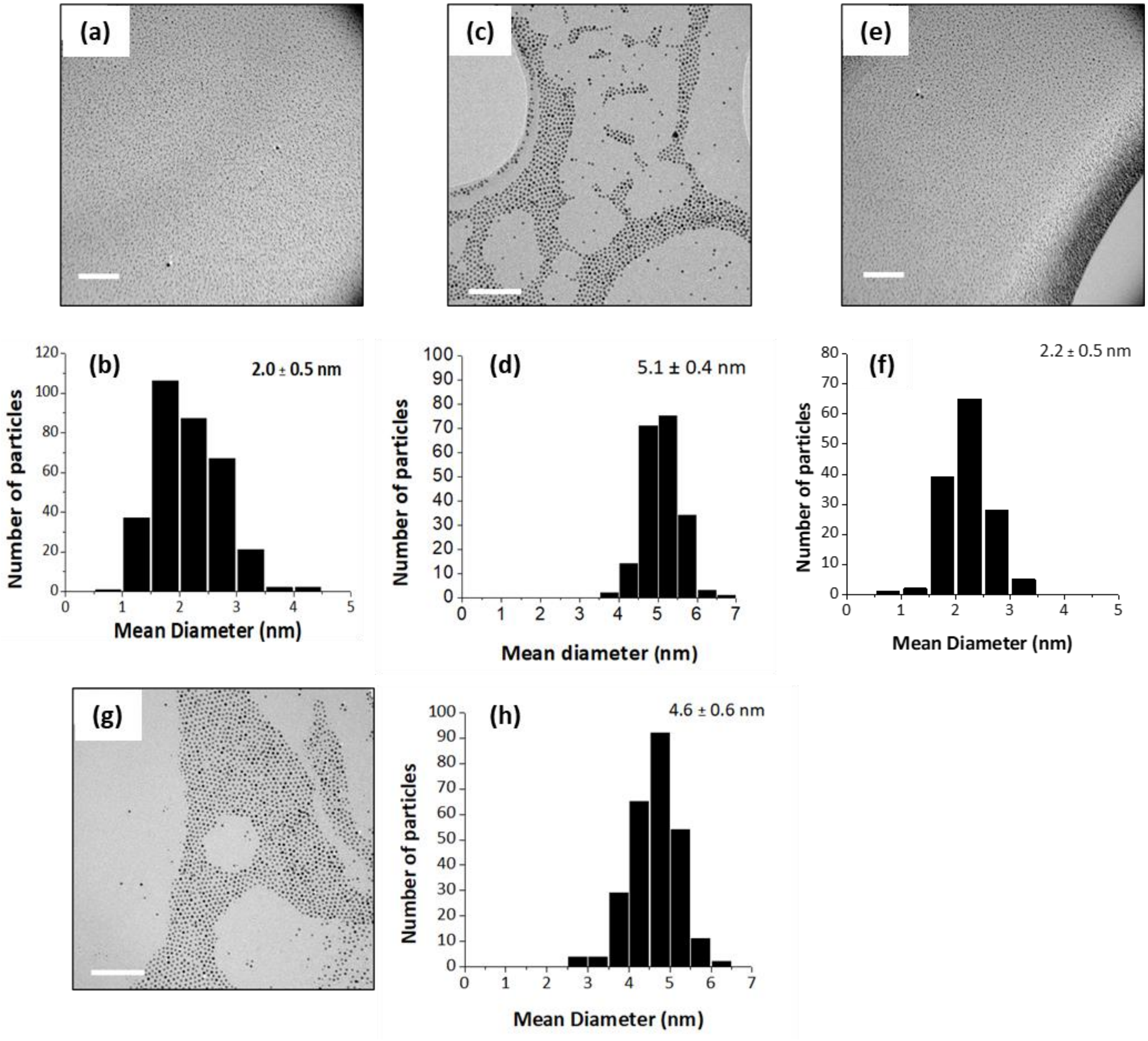

Figure S1: *NP´s micrographs and size distribution histograms of the nanoparticles (a-b)* ***AuDTL2 0 minutes****, (c-d)* ***AuDTL2 180 minutes****, (e-f)* ***AuDTL3 0 minutes****, (g-h)* ***AuDTL3 180 minutes*** *determined by Scanning Transmission Electron Microscopy.*

1.2 TGA studies of AuDTLn.

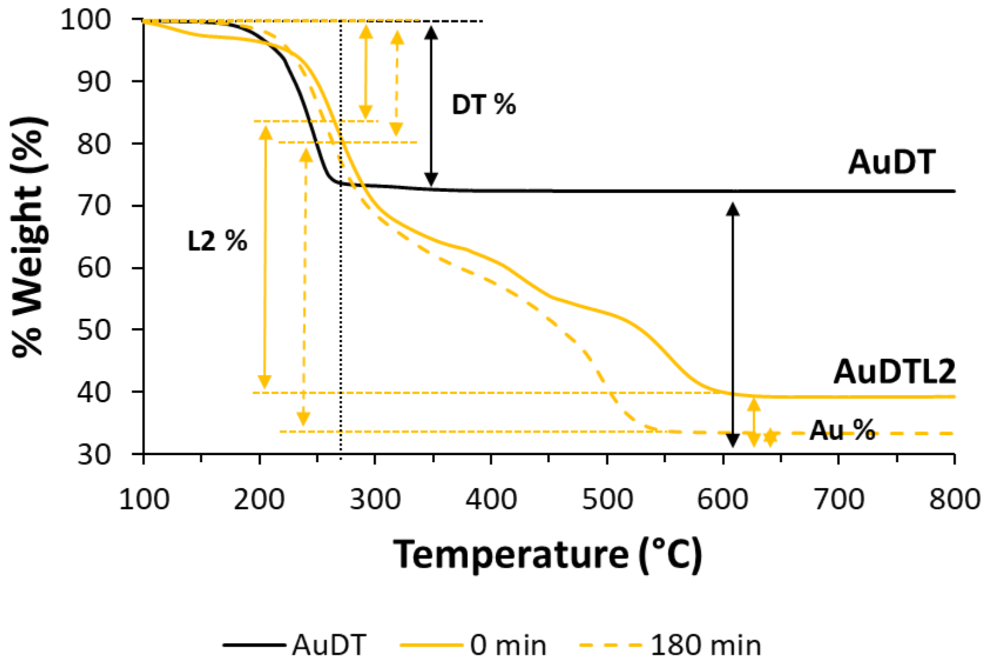

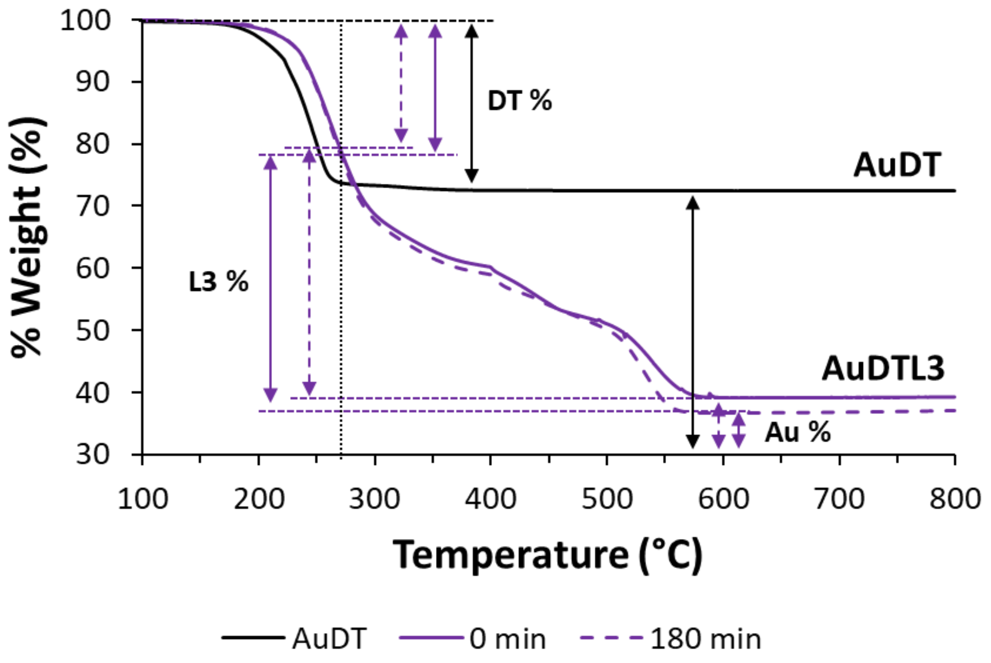

Figure S2: *TGA data analysis proportions of inorganic/metallic core and organic proportion in each sample obtained after different treatment times: 0 minutes (continuous line) and 180 minutes (dashed line).*

1.3 Sample holder design and properties

The samples of dendrimer-coated particles are characterized by very small values of the magnetic moment. Therefore, it was of paramount importance to design and employ a sample holder capable of reducing the background signal as much as possible, thus enabling to obtain the sample magnetization directly, that is, avoiding error-prone deconvolution operations.

Conventionally, the sample holder consists of a (gelatin) capsule fixed inside a (polypropylene) straw. This type of holder is excellent because of easy manipulation and preparation but requires the signal of the sample to be much larger than that of the holder. Its disadvantage lies in that the end of the capsule, just below the sample, also contributes to the overall signal, resulting in an induced voltage response with asymmetric shape. Therefore, the uncertainty in the "centering position" of the sample with respect to the pick-up coils leads to a loss of sensitivity, and to the need of subtracting the holder signal before deconvolution of the measured signal, which is not a simple operation. Failing to do so or, worse, ignoring the effect altogether by directly subtracting the diamagnetism of the capsule measured separately, might result in unreliable data, the more so the smaller the magnetic signal that one wants to detect.

A homogenously magnetized holder, moving as a whole through the pick-up coils, does not induce a voltage at all. Our approach exploits this effect. We make use of a hollow quartz tube, wherein the sample can be fixed by sliding inside two cylinders, or pistons. The material used for the tube and pistons is suprasil, which is a very homogenous quartz glass with the smallest degree of magnetic contaminations available. The measured magnetization consists of the signal due to the sample and a signal due to the volume of suprasil that is missing at the position of the sample, i.e., $M_{\mathrm{meas}} = M_{\mathrm{sample}} - M_{\mathrm{quartz}}$ (hence explaining, for diamagnetic quartz, the positive slope of the magnetization of the empty sample holder in Fig. 3). Importantly and in contrast to the conventional capsule-straw approach, the signal induced here by the holder and the sample are exactly at the same position and of the same shape. Therefore, the magnetization of the sample holder can be determined separately, for a slit volume between the two pistons equivalent to the volume occupied by the sample, then can be subtracted from the total signal without doing any harm. The measurement of the empty holder provides a diamagnetic response of the quartz, a straight line in field (see, e.g., Fig. 3), which is linearly proportional to the distance between the pistons.
